# A new hybrid bismuth chloride semiconductor (C_5_H_7_BrN_3_)_6_(BiCl_5_)_3_: powder XRD, optical properties, and DFT investigation

**DOI:** 10.1039/d5ra09478j

**Published:** 2026-01-30

**Authors:** Chaima Jridi, Nour Elleuch, Sergiu Shova, Jerome Lhoste, Frédéric Amiard, Mohamed Boujelbene

**Affiliations:** a Laboratory of Physico-Chemistry of Solid State, LR11ES51, Sfax Faculty of Sciences, University of Sfax Sfax 3071 Tunisia m_boujelbene2010@yahoo.fr; b “Petru Poni” Institute of Macromolecular Chemistry Alea Grigore Ghica Voda 41-A 700487 Iasi Romania; c MMM-UMR 6283 CNRS, Lunam, Faculty of Sciences and Techniques, University of Maine Avenue Olivier Messiaen 72085. Le Mans Cedex 9 France

## Abstract

In this work, we report the synthesis and characterization of the new hybrid inorganic–organic compound (C_5_H_7_BrN_3_)_6_(BiCl_5_)_3_, which crystallizes in the triclinic space group *P̄*1. The crystal structure, determined from X-ray powder diffraction, reveals an asymmetric unit built from one [Bi_2_Cl_10_]^4−^ dimer, one independent [BiCl_5_]^2−^ anion, and six neutral 2,3-diamino-5-bromopyridinium molecules. This arrangement highlights the coexistence of distinct bismuth chloride units stabilized by an extensive network of hydrogen bonding and electrostatic interactions with the organic moieties. Density Functional Theory (DFT) calculations performed at the B3LYP/LANL2DZ level provide optimized structural parameters and vibrational assignments consistent with the experimental IR spectrum recorded in the 400–4000 cm^−1^ range. The optical properties investigated *via* solid-state UV-vis diffuse reflectance reveal an indirect bandgap of 2.6 eV, confirming the semiconducting behavior of the material. The combination of structural features, optical response, and theoretical insights underscores the potential of (C_5_H_7_BrN_3_)_6_(BiCl_5_)_3_ as a promising hybrid material for semiconductor and optoelectronic applications.

## Abstract 2

In this work, we report the synthesis and characterization of the new hybrid inorganic–organic compound (C_5_H_7_BrN_3_)_6_(BiCl_5_)_3_, which crystallizes in the triclinic space group *P̄*1.

## Graphical abstract

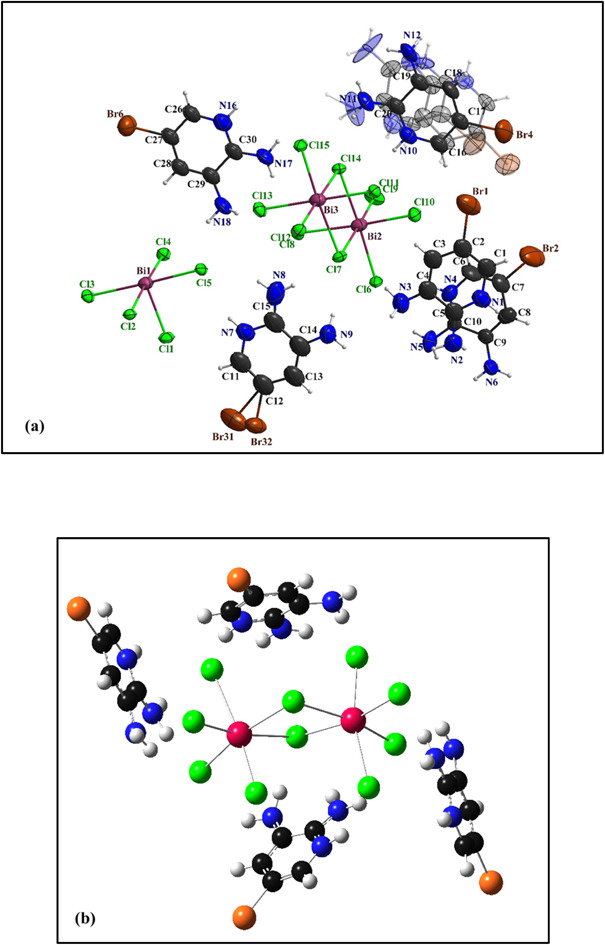

## 1. Intoduction

Hybrid organic–inorganic materials continue to attract growing attention due to their ability to merge the structural versatility of organic molecules with the robust physicochemical properties of inorganic frameworks. This synergy often leads to emergent functionalities that outperform those of purely organic or inorganic systems, particularly in domains such as catalysis, optoelectronics, nonlinear optics, energy storage, and environmental remediation.^1–3^ The tunability of these hybrid materials arises not only from the modularity of the inorganic building units but also from the vast diversity of organic cations that can be engineered to tailor intermolecular interactions, electronic properties, and structural stability.

Among inorganic components, bismuth-based motifs have emerged as promising candidates due to the unique characteristics of bismuth, including its relatively low toxicity, strong spin–orbit coupling, high atomic number, and rich coordination chemistry.^4^ Bismuth-containing hybrids have demonstrated remarkable performance in photocatalysis, especially under visible-light irradiation, and have been explored for energy conversion, radiation shielding, electrochemical storage, and biomedical applications. Halobismuthate complexes, in particular, have gained renewed interest as potential lead-free alternatives to halide perovskites, offering improved environmental compatibility while retaining advantageous optical and electronic features.

Parallel to the evolution of inorganic components, significant effort has been invested in identifying functional organic cations capable of enhancing the stability and performance of hybrid frameworks. Aromatic heterocycles bearing electron-donating or electron-withdrawing substituents are especially valuable, as their electronic anisotropy and coordination behavior can markedly influence crystal packing, charge distribution, and supramolecular interactions. Within this context, 2,3-diamino-5-bromopyridinium, derived from a pyridinium core functionalized with two amino groups and a bromine atom, represents a particularly attractive building block. The amino groups promote hydrogen bonding and flexibility in supramolecular assembly, while the bromine atom increases molecular polarizability and may contribute to enhanced optical responses. Despite these favourable features, hybrid materials incorporating this cation remain largely unexplored in the literature, highlighting a significant opportunity for innovation.

In this work, we report the synthesis and structural characterization of a new halobismuthate hybrid compound, (C_5_H_7_BrN_3_)_6_(BiCl_5_)_3_, based on the 2,3-diamino-5-bromopyridinium cation. The combination of this highly functionalized organic species with the BiCl_5_^2−^ inorganic units provides a relevant platform for exploring structure–property relationships within bismuth-based hybrid systems. To gain comprehensive insight into its physicochemical behavior, the compound was investigated using optical measurements, infrared spectroscopy, and (DFT) calculations, complemented by powder X-ray diffraction and energy-dispersive X-ray (EDX) analysis. The results contribute to expanding the family of hybrid halobismuthates and open promising perspectives for the development of functional materials with potential optoelectronic and photophysical applications.

## 2. Experimental characterisation techniques

The experimental and computational techniques employed for the synthesis and characterization of the hybrid compound (C_5_H_7_BrN_3_)_6_(BiCl_5_)_3_ are described below.

### 2.1. Chemical preparation

The new hybrid material was obtained using a hydrothermal synthesis method. This technique offers several advantages, including the ability to grow high-quality crystals under controlled temperature and pressure conditions, as well as to stabilize metastable phases. In our procedure, 2,3-diamino-5-bromopyridine (97%) and bismuth(iii) chloride (99%) were dissolved in distilled water at a molar ratio of 2 : 1. The resulting solution, containing the preformed chemical species, was then transferred into a Teflon-lined autoclave, after which a few drops of concentrated hydrochloric acid (38% purity) were added. The autoclave was sealed and placed in a hydrothermal oven, where the mixture was maintained at 150 °C for four days under autogenous pressure to promote nanocrystal formation. After cooling, brown crystals were obtained, and a single crystal suitable for X-ray structural analysis was carefully selected.

### 2.2. X-ray data collection

A brown crystal was selected for analysis using an Oxford-Diffraction XCALIBUR E CCD diffractometer equipped with graphite-monochromated Mo Kα radiation (*λ* = 0.71073 Å). Data were collected at 293 K. The structure was solved with SHELXT-2018/2 and refined by full-matrix least-squares methods on F2 using SHELXL-2018/3,^5–7^ operated through the Olex2 interface^8^ within the SHELX suite implemented in WinGX.^9^ Refinement converged with final *R* and *wR* values of 0.066 and 0.149, respectively, using anisotropic displacement parameters for all non-hydrogen atoms. Hydrogen atoms were positioned geometrically and refined using a riding model. Comprehensive crystallographic and refinement details are provided in (Table 1).

**Table 1:** Crystal data and structure refinement details of (C_5_H_7_BrN_3_)_6_(BiCl_5_)_3_

Crystal data	
Formula	(C_5_H_7_BrN_3_)_6_(BiCl_5_)_3_
Formula weight (g mol^−1^)	2292.96
Density calc (Mg m^−3^)	2.414
Color	Brown
Crystal system	Triclinic
Space group	*P̄*1
*a*(Å)	10.9543 (5)
*b*(Å)	16.4575 (7)
*c* (Å)	19.2722 (8)
*α*	114.469 (4)°
*β*	92.108 (4)°
*γ*	92.067 (4)°
Z	2
Volume (Å^3^)	3155.2 (3)
Radiation du Mo	Mo Kα
Diffractometer	XCALIBUR E CCD
Crystal size (mm^3^)	0.15 × 0.08 × 0.04
Temperature (*K*)	293
Wavelength (Å)	0.71073
Absorption coefficient µ (mm^−1^)	12.82
Limiting indices	−13 ≤ *h* ≤ 13
	−19 ≤ *k* ≤ 18
	−21 ≤ *l* ≤ 22
Reflections collected	26 631
Independent reflections	11 118
*θ* range for data collection (°)	2.1 < *θ* < 25
Reflections with I > 2*σ*(I)	5905
F(000)	2124
Number of refined parameters	610
Residual fourier density (e Å^−3^)	−1.64<Δ*ρ*< 1.93
*wR* (*F*^2^)	0.149
*R* [*F*^2^ > 2*σ*(*F*^2^)]	0.066
S = GooF	1.01
CCDC	2495900

### 2.3. Micrographs and X-ray microanalysis

An Oxford X-Max EDX microanalysis system coupled with a JEOL-6610LV scanning electron microscope (JEOL Ltd, Japan) operating at 20 kV was employed to characterize the crystal morphology.

### 2.4. FT-IR spectroscopy

Fourier-transform infrared (FT-IR) spectra were recorded at room temperature using a Bruker Vertex 70V spectrometer, over the 400–4000 cm^−1^ range, with a spectral resolution of 4 cm^−1^ and by averaging 40 scans.

### 2.5. UV-vis measurements

UV-vis optical measurements were performed at room temperature using a Shimadzu UV-visible-NIR spectrophotometer operating over the 200–1100 nm wavelength range. Absorbance (*A*) values were calculated from the recorded reflectance data.

### 2.6. Computational details

All calculations were carried out using the Gaussian 09 software package.^10^ The ground-state geometry was optimized within the framework of (DFT) employing the B3LYP functional and the LANL2DZ basis set. A vibrational frequency analysis was subsequently performed on the optimized geometry to verify the absence of imaginary frequencies, thereby confirming that the structure corresponds to a true global minimum.

## 3. Results and discussion

### 3.1. Structure description of (C_5_H_7_BrN_3_)_6_(BiCl_5_)_3_

**Fig. 1:**
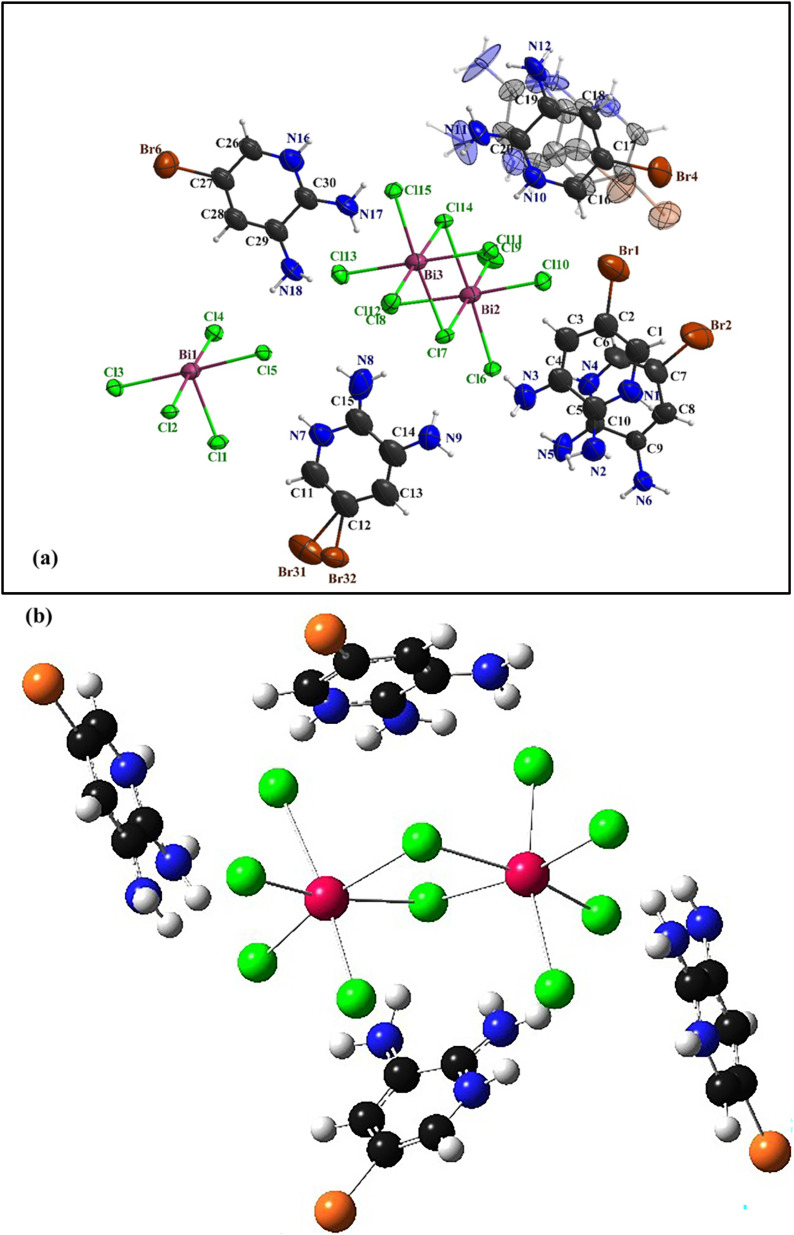
(1a) The asymmetric unit of the (C_5_H_7_BrN_3_)_6_(BiCl_5_)_3_. (1b) Optimized molecular structure with DFT/B3LYP method and LANL2DZ basis set.

**Fig. 2:**
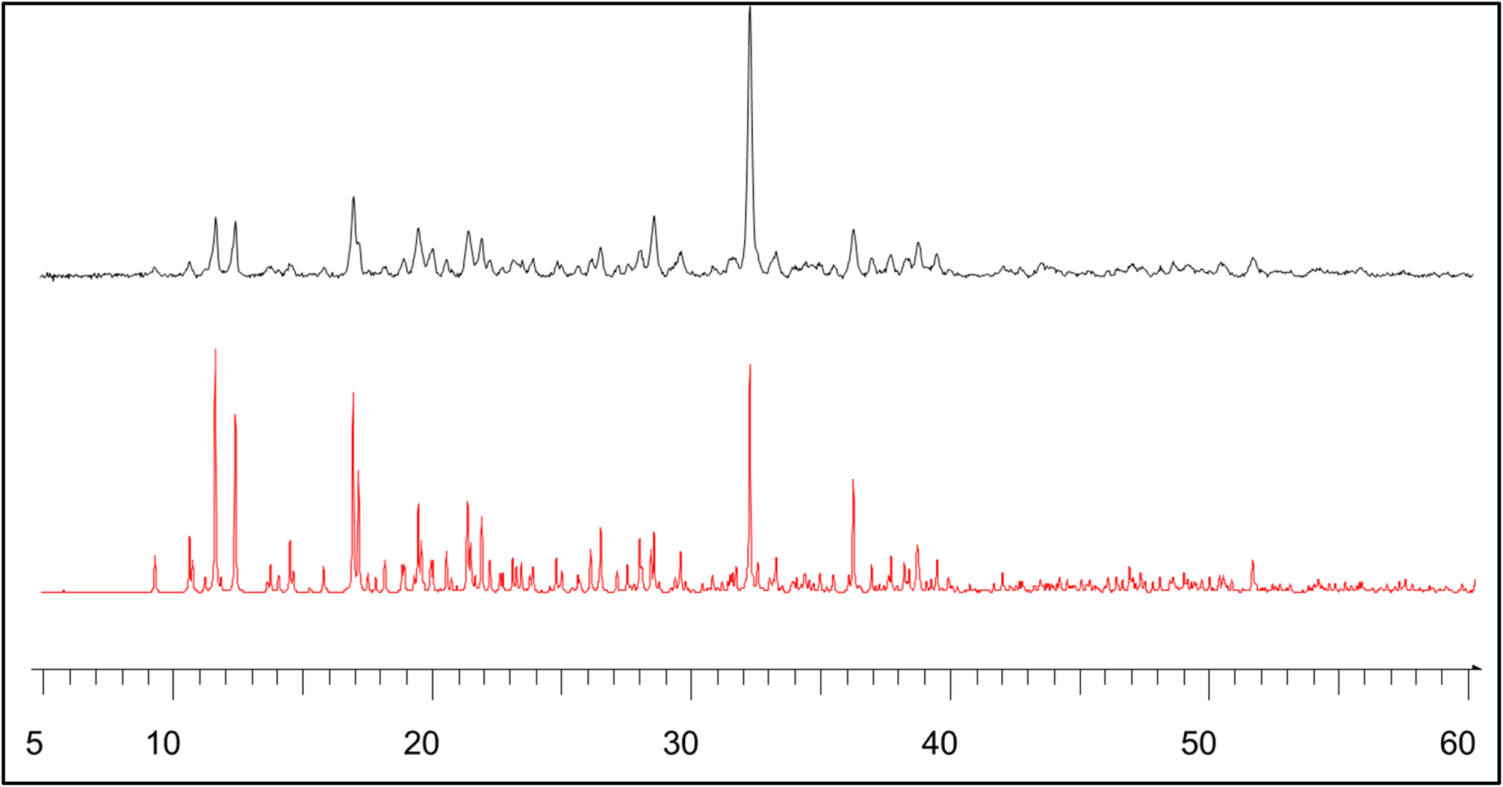
Comparison of calculated and experimental PXRD patterns for (C_5_H_7_BrN_3_)_6_(BiCl_5_)_3_.

The compound (C_5_H_7_BrN_3_)_6_(BiCl_5_)_3_ crystallizes in the triclinic system, space group *P̄*1, with the asymmetric unit consisting of one [Bi_2_Cl_10_]^4−^ dimer, one independent [BiCl_5_]^2−^ anion, and six neutral 2,3-diamino-5-bromopyridinuim molecules (Fig. 1a). The optimized geometry of the title compound is presented in (Fig. 1b). The [Bi_2_Cl_10_]^4−^ dimer is built from two edge-sharing [BiCl_6_] octahedra, while the monomeric [BiCl_5_]^2−^ unit remains isolated. A positional disorder is found only in the organic part. Some bromine atoms and amino groups of the 2,3-diamino-5-bromopyridinuim molecules can occupy two nearby positions, each partially filled. This disorder shows that the ligands have a small freedom to rotate within the crystal. It was modeled using split atomic positions and adjusted occupancy factors.^11^ The phase purity and crystallographic structure of (C_5_H_7_BrN_3_)_6_(BiCl_5_)_3_ were examined by powder X-ray diffraction (PXRD). Fig. 2 shows the comparison between the experimental PXRD pattern and the simulated pattern obtained from single-crystal X-ray diffraction data. A good agreement between the peak positions confirms the phase purity and structural integrity of the compound. The major diffraction peaks observed at 2*θ* ≈ 11.12°, 15.84°, 18.43°, 22.61°, 28.94°, and 32.10° were indexed to the triclinic crystal system with space group *P̄*1. The sharp and well-defined peaks indicate good crystallinity and the absence of secondary phases. The crystallite size (*D*) was calculated using the Debye–Scherrer equation:
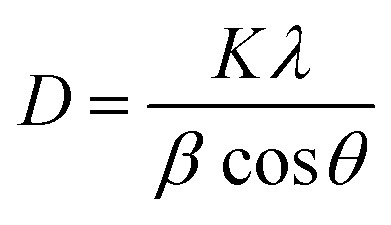
where *K* = 0.9, *λ* = 1.5406 Å (Cu Kα radiation), *β* is the full width at half maximum (FWHM) in radians, and *θ* is the Bragg angle. The calculated crystallite sizes range from ∼38 to 46 nm, with an average crystallite size of 42 ± 4 nm. The dislocation density (*δ*), which represents the amount of crystallographic defects, was calculated using:
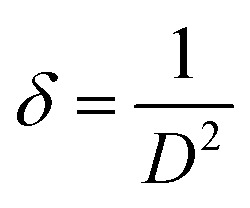


The lattice micro-strain (*ε*) was evaluated using the relation:
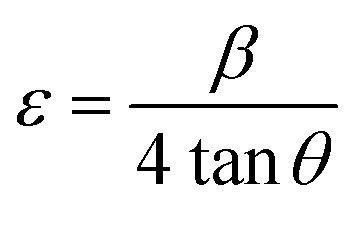


The calculated micro-strain values are on the order of 10^−3^, indicating low lattice distortion and minimal internal stress. Such low defect density and strain confirm the high structural quality of the material. This crystallographic order is particularly important for zero-dimensional (0D) hybrid semiconductors, where electronic and optical properties are governed by localized inorganic units such as [BiCl_5_]^2−^ and [Bi_2_Cl_10_]^4−^ clusters (Table 2).^12^

**Table 2:** XRD structural parameters of (C_5_H_7_BrN_3_)_6_(BiCl_5_)_3_

Peak position (2*θ*, °)	FWHM (°)	*D* (nm)	*δ* (nm^−2^)	*δ* (×10^−3^ nm^−2^)	Microstrain *ε*	*ε* (×10^−3^)
11.12	0.38	46.2	0.00047	0.47	0.0029	2.9
15.84	0.44	41.8	0.00057	0.57	0.0034	3.4
18.43	0.51	38.9	0.00066	0.66	0.0038	3.8
22.61	0.47	40.6	0.00061	0.61	0.0032	3.2
28.94	0.36	44.7	0.00050	0.50	0.0026	2.6
32.10	0.41	39.8	0.00063	0.63	0.0031	3.1

**Fig. 3:**
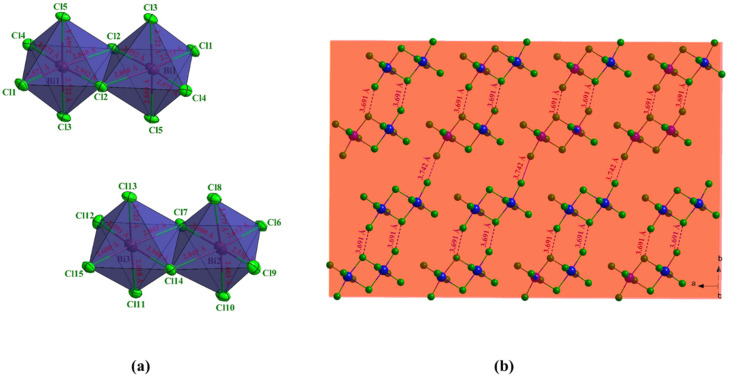
Bond distances (° A) for polyhedron (a), short Cl⋯Cl interactions (b).

**Fig. 4:**
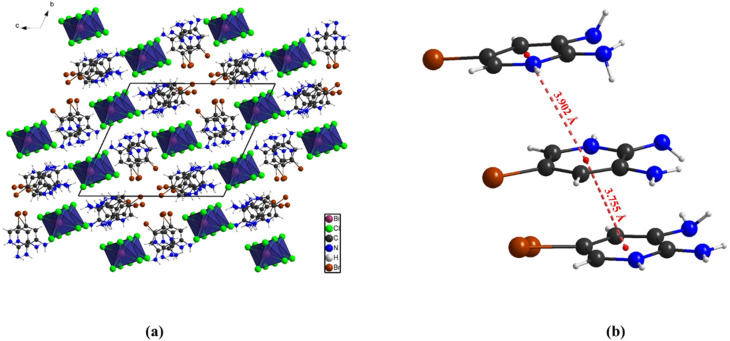
Projection of the (C_5_H_7_BrN_3_)_6_(BiCl_5_)_3_ structure in the plane (b, c) (a), π–π interactions in the crystal structure (b).

Each Bi^3+^ ion within the [Bi_2_Cl_10_]^4−^ dimer is coordinated by two bridging and four terminal chloride ligands, forming a distorted octahedral geometry. The Bi–Cl bond lengths range from 2.572(4) to 3.000(4) Å, with the Bi–Cl terminal bonds being significantly shorter than the Bi–Cl bridging ones, indicating a noticeable asymmetry in the coordination environment. In addition to the dimeric unit, the structure also contains an independent [BiCl_5_]^2−^ anion, where the Bi center adopts a distorted octahedral coordination with one less occupied site due to the influence of the 6 s^2^ lone pair. The distortion indices (*D*) of the three crystallographically independent octahedra are 0.044 for Bi_1_, 0.052 for Bi_2_, and 0.042 for Bi_3_, respectively (Fig. 3a).^13^ The octahedra are connected through shared chloride bridges to form [Bi_2_Cl_10_]^4−^ dimers, in which the Cl⋯Cl separations are 3.691 and 3.742 Å (Fig. 3b). These dimers align along the crystallographic *b* axis, generating extended chains of edge-sharing polyhedra. The projection along the *c* axis reveals an ordered alternation of inorganic sheets and organic moieties (Fig. 4a). The aromatic cations are arranged in parallel, forming π–π stacking interactions between adjacent pyridinium rings with centroid-to-centroid distances of approximately 3.90 and 3.75 Å (Fig. 4b).^14^ In addition, the crystal structure is further stabilized by N–H⋯Cl hydrogen bonds linking the organic cations to the inorganic anionic framework, thus reinforcing the overall three-dimensional supramolecular architecture (Table 3).

**Table 3:** Geometry of hydrogen bonds for [C_5_H_6_ClN_2_]_2_BiCl_5_^a^

	D-H (Å)	H⋯A (Å)	D⋯A (Å)	∠D-H⋯A(°)
N13A-H13A⋯Cl3v	0.86	2.42	3.26(3)	167
N17–H17A⋯Cl15iv	0.86	2.51	3.295(13)	153
N7–H7⋯Cl5	0.86	2.46	3.272(17)	157
N13B–H13B⋯Cl1^v^	0.86	2.44	3.14(3)	140

a Symmetry codes: −*z*; (iv) −*x*, −*y*+2, −*z*; (v) *x*, *y*+1, *z*+1.

### 3.2. EDX and elemental mapping analysis

**Fig. 5:**
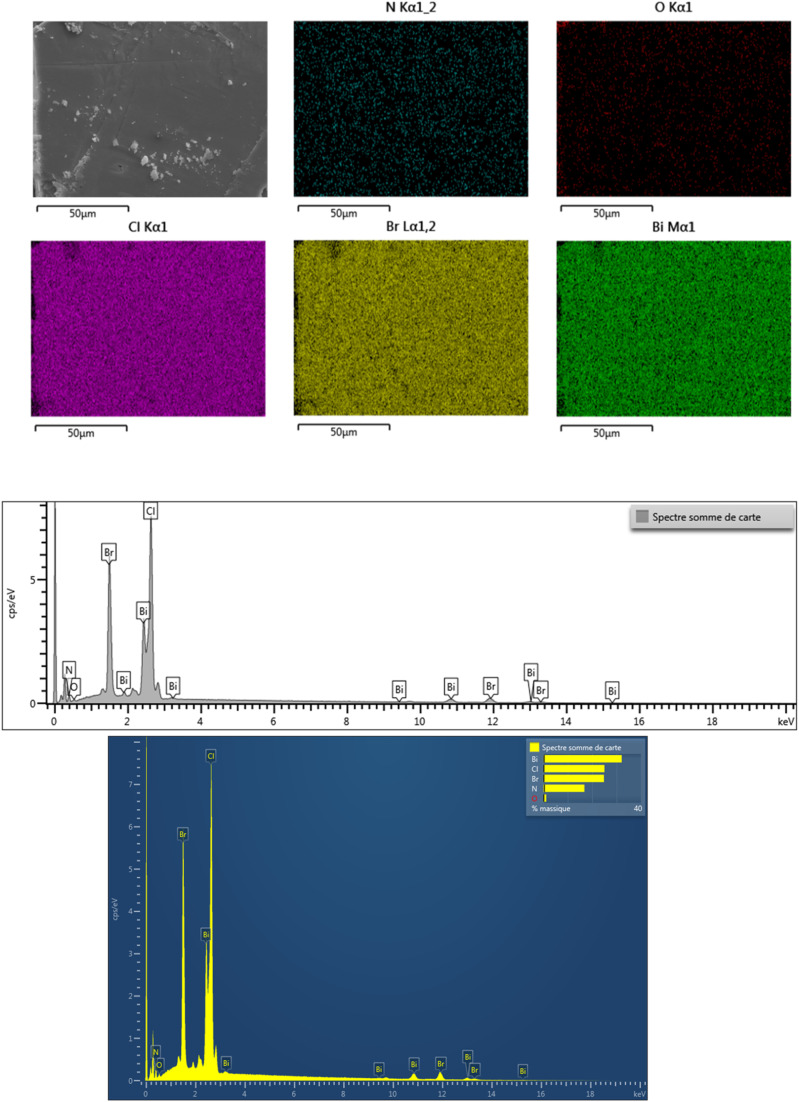
EDX Spectra and elemental maps obtained by scanning electron microscopy.

The EDX elemental mapping confirms that the elements expected in the compound (C_5_H_7_BrN_3_)_6_(BiCl_5_)_3_ are well distributed across the surface. The maps show a homogeneous presence of nitrogen, chlorine, bromine, and bismuth, indicating good chemical uniformity in the material. Oxygen appears in a very small amount, which may be due to slight surface oxidation or environmental exposure. The corresponding EDX spectrum further supports this result, with clear peaks for N, Cl, Br, and Bi. The atomic percentages (49.16% N, 28.91% Cl, 12.72% Br, and 6.29% Bi) are consistent with the expected composition of the hybrid framework. These results confirm that the synthesized material contains all the targeted elements and that they are evenly distributed, demonstrating successful formation of the bismuth-based hybrid compound (Fig. 5).

### 3.3. FT-IR spectra

**Fig. 6:**
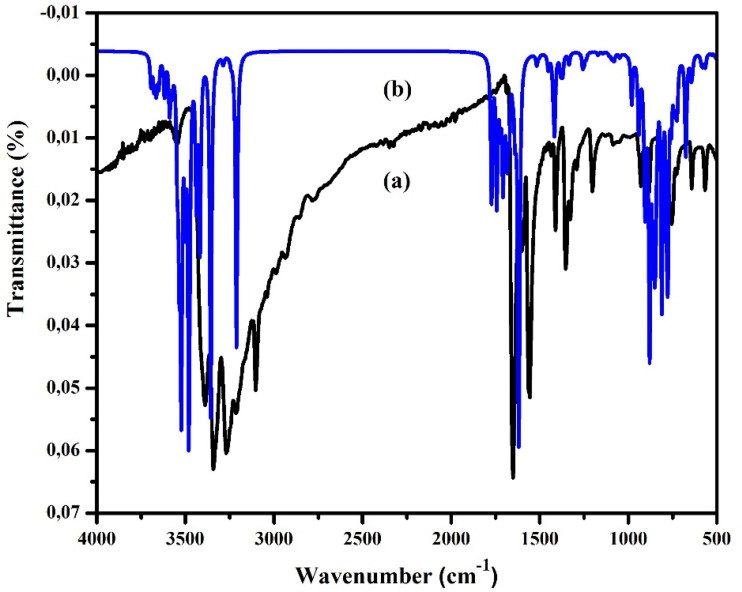
Room temperature experimental (a) and theoretical (b) IR spectra of (C_5_H_7_BrN_3_)_6_(BiCl_5_)_3_.

The infrared spectrum of the compound (C_5_H_7_BrN_3_)_6_(BiCl_5_)_3_, recorded in the range 500–3600 cm^−1^ (Fig. 6), exhibits several characteristic vibrational bands consistent with the functional groups present in the organic cation. The strong absorptions at 3547, 3389, and 3341 cm^−1^ are assigned to the asymmetric and symmetric stretching modes of the NH_2_ group, whereas the band at 3268 cm^−1^ corresponds to the N–H stretching vibration. The peak at 3101 cm^−1^ is attributed to the aromatic C–H stretching mode. In the mid-IR region, the absorption observed at 1651 cm^−1^ is associated with the N–H bending vibration, while the bands at 1597 and 1560 cm^−1^ correspond to C–H in-plane bending modes of the heteroaromatic ring. The C=C stretching vibration appears at 1407 cm^−1^, and the band recorded at 1350 cm^−1^ is assigned to the torsional deformation of the NH_2_ group, *τ*(NH_2_). The absorption at 1289 cm^−1^ is attributed to the C–N stretching mode. At lower wavenumbers, the peaks at 1203 and 929 cm^−1^ are attributed to out-of-plane C–H bending vibrations, while the band at 874 cm^−1^ corresponds to the NH_2_ twisting mode, *ω*(NH_2_). The deformation of the N–C–C fragment is identified at 752 cm^−1^, followed by the C–Br stretching vibration at 641 cm^−1^. The low-frequency band at 567 cm^−1^is attributed to the C–C–C skeletal deformation. The good agreement between the experimental and calculated vibrational frequencies (Table 4) supports the proposed structural features of the organic cation within the (C_5_H_7_BrN_3_)_6_(BiCl_5_)_3_ framework.^15,16^

**Table 4:** The attributions of calculated and observed frequencies of the vibration modes of the compound^a^

Obs. wavenumbers (cm^−1^)	Calc. wavenumbers (cm^−1^)	Assignment
3547	3563	*υ*_as_(NH_2_)
3389	3383	*υ*_s_(NH_2_)
3341	3322	*υ*_s_(NH_2_)
3268	3262	*ν*(N–H)
3101	3115	*ν*(C–H)
1651	1668	δ(N–H)
1597	1590	β(N–H)
1560	1531	β(C–H)
1407	1419	*ν*(C–C)
1350	1327	*δ*(C–H)
1289	1276	*τ*(NH_2_)
1203	1177	*ν*(C–N)
929	964	γ(C–H)
874	884	*ω*(NH_2_)
752	760	*δ*(N–C=C)
641	626	*ν*(C–Br)
567	578	*δ*(C–C=C)

a *υ*_s_: symetric stretching, *υ*_as_: assymetric stretching, β: in plane bending, γ: out plane bending, *δ*: scissoring, *ω*: wagging, *τ*: twisting.

### 3.4. Reduced density gradient (RDG), ELF and LOL analyses

**Fig. 7:**
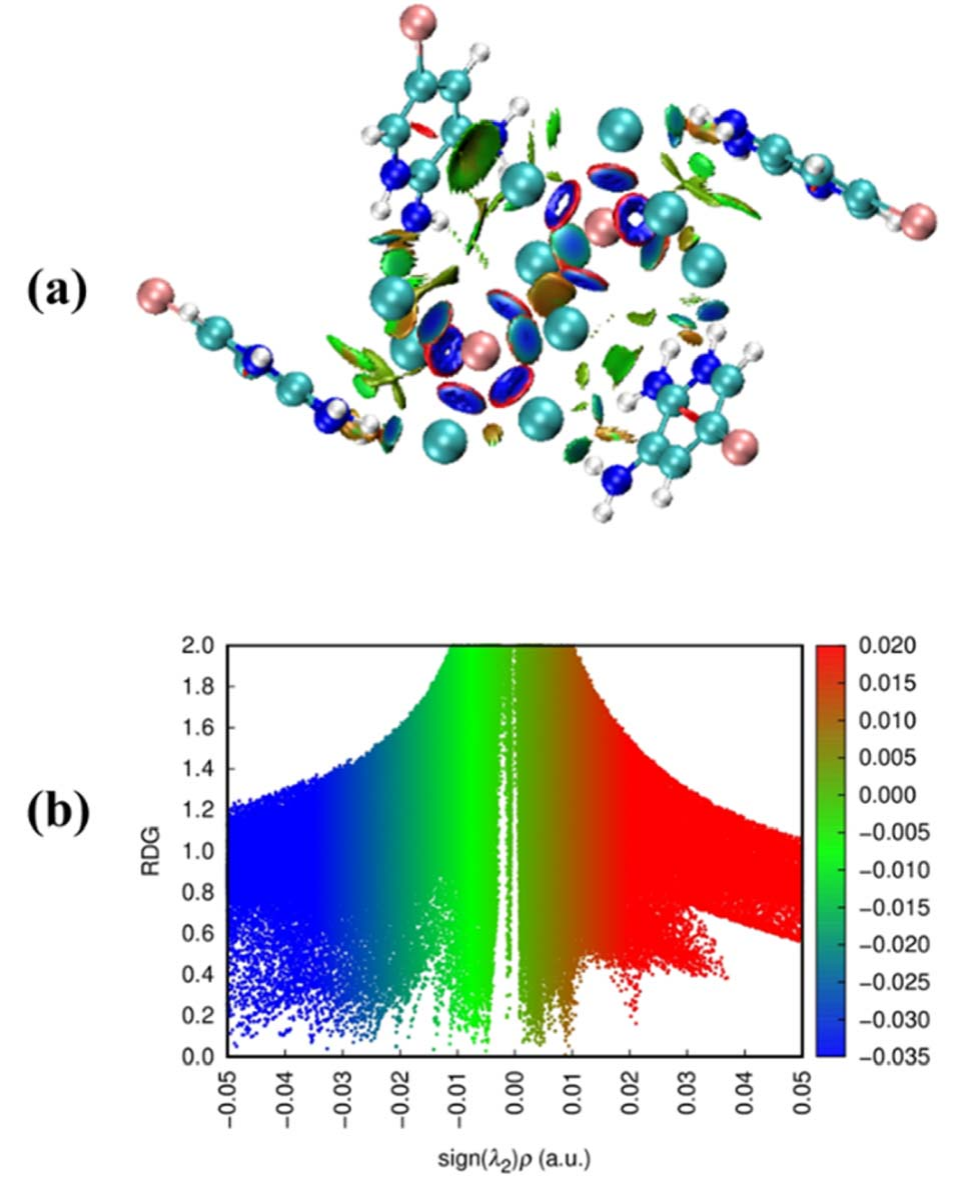
Representation of different types of interactions in (C_5_H_7_BrN_3_)_6_(BiCl_5_)_3_ compound (a).the map of Reduced Density Gradient (RDG) defines the interaction limits for (C_5_H_7_BrN_3_)_6_(BiCl_5_)_3_ (b).

Intermolecular interactions is identified using an experimental single-crystal X-ray diffraction, supported by geometric optimization. These interactions were further characterized by multi-wavefunction analysis through reduced density gradient (RDG) plots. The RDG method, introduced by Johnson *et al.*,^17^ is a dimensionless descriptor derived from the electron density and its first derivative, enabling a detailed assessment of noncovalent interactions (Fig. 7).
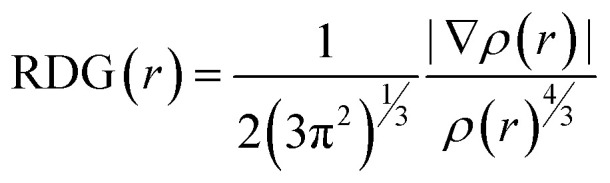


The RDG analysis of the compound (C_5_H_7_BrN_3_)_6_(BiCl_5_)_3_ reveals three distinct interaction regions located in low-density zones, characterized by reduced RDG values. The nature of these interactions within the crystalline framework was examined through the relationship between the electron density *ρ*(*r*) and the parameter *λ*_2_. As in typical NCI (Non-Covalent Interactions) analyses, these regions are represented by blue, green, and red features.

The red regions correspond to steric repulsion, appearing where the condition (*λ*_2_)*ρ* > 0 is satisfied. The green areas indicate van der Waals interactions, associated with (*λ*_2_)*ρ* ≈ 0. The blue spikes, observed in the negative (*λ*_2_)*ρ* domain, reflect strong attractive forces such as hydrogen bonding.

In this system, the green and bluish plates found between the N–H donors and chloride acceptors clearly highlight the presence of N–H⋯Cl hydrogen bonds, confirming their attractive nature and their contribution to the stabilization of the crystal structure. Furthermore, the red regions located around the aromatic rings reflect steric effects within the molecular packing. To evaluate the intensity and spatial distribution of these intermolecular interactions, the RDG *versus* sign(*λ*_2_)*ρ* plot was constructed. This color-coded representation provides a detailed visualization of all interaction zones in three-dimensional space, offering direct insight into the strength and distribution of the N–H⋯Cl hydrogen bonds in the lattice.

**Fig. 8:**
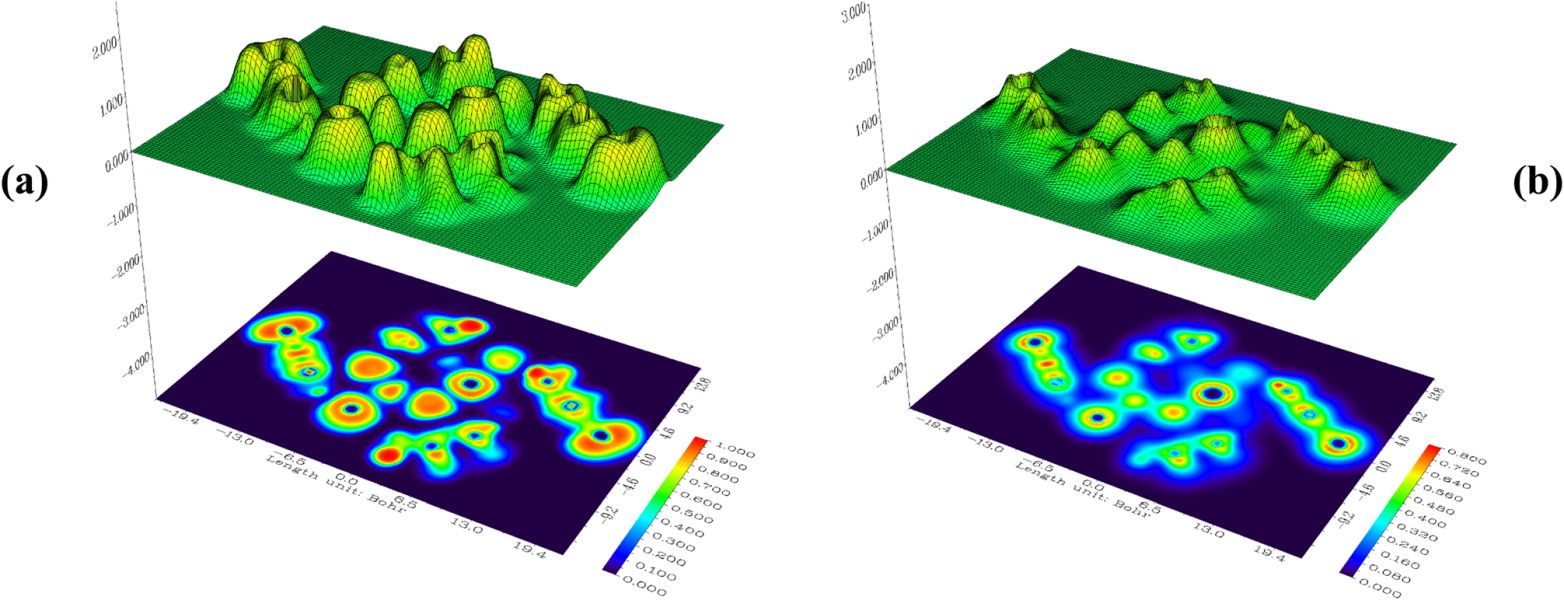
Electron localization function (ELF) maps (a), localized orbital locator (LOL) maps (b).

Surface analysis of the compound (C_5_H_7_BrN_3_)_6_(BiCl_5_)_3_ was carried out using the Electron Localization Function (ELF), complemented by Relief maps and LOL maps (Fig. 8). The relief maps, displaying prominent or reduced peaks, provide detailed insight into the electron environment around each atom, indicating the probability of finding an electron pair at the molecular surface. Although both ELF and LOL are based on the kinetic energy density, they differ in methodology. ELF is derived from electron pair density, highlighting regions where electron pairs are highly localized. In contrast, LOL maps illustrate the gradient of localized orbitals and are especially useful in regions where these orbitals overlap. The ELF and LOL images of (C_5_H_7_BrN_3_)_6_(BiCl_5_)_3_ are displayed using a color shade map, along with contour maps for hydrogen-bonding regions. The ELF scale ranges from 0.0 to 1.0, with delocalized electrons typically appearing in the lower range (<0.5). In areas where the electron density is mainly influenced by electron positions, LOL values exceed 0.5, reflecting higher electron localization. In this compound, the electron cloud shows delocalization around certain carbon atoms within the organic ligands, represented by blue regions in the maps. ELF maps clearly reveal critical points and their trajectories, as well as chemically significant regions (in red and orange), which are mainly localized around hydrogen atoms and consistent across the studied units. Central regions of some hydrogen atoms appear white, indicating that the electron density exceeds the upper limit of the color scale (0.80). Compared to ELF, the LOL maps provide a more vibrant and decisive depiction of electron distribution, offering clearer insights into electron localization and interaction regions, particularly around the organic moieties and the bismuth–chloride coordination environment. These maps help visualize areas of covalent bonding, electron delocalization, and potential interaction sites within the complex.^18,19^

### 3.5. UV-vis diffuse reflectance study

#### 3.5.1. Determination of the band-gap energy

**Fig. 9:**
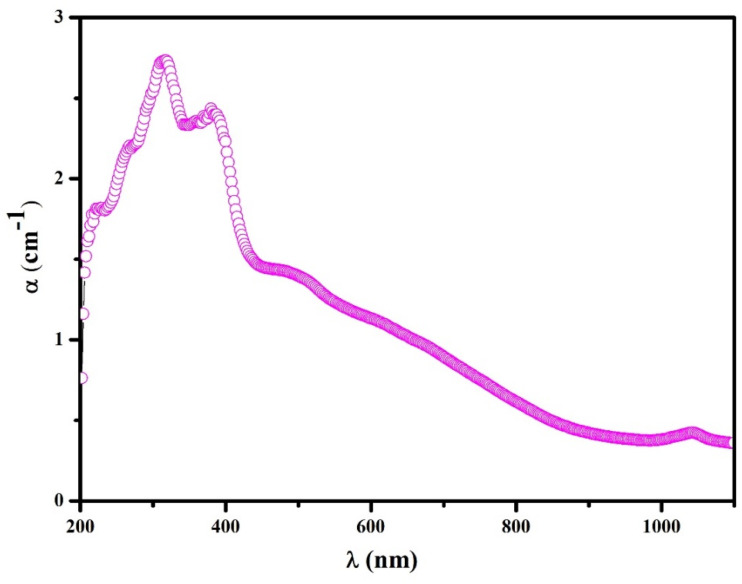
Evolution of the absorption coefficient *α* of the (C_5_H_7_BrN_3_)_6_(BiCl_5_)_3_compound according to the wavelength *λ*.

The absorption coefficient is an important parameter because it shows how well a material can absorb light, which is useful for solar and optoelectronic applications.^20^ The absorption spectrum of the (C_5_H_7_BrN_3_)_6_(BiCl_5_)_3_ compound, shown in Fig. 9, was obtained from UV-vis absorbance data using the relation *α* = 2.303A d, where *A* is the absorbance and *d* is the sample thickness (1 mm). The absorption coefficient *α* was evaluated in the wavelength range from 200 to 1100 nm. The material absorbs light strongly in the UV and visible regions, reaching values close to 3.0 cm^−1^ at short wavelengths. Several clear absorption peaks appear between 250 and 350 nm, which are mainly due to electronic transitions inside the organic part of the structure and to charge-transfer processes between chloride ions and Bi(iii).^21,22^ After 400 nm, the absorption decreases smoothly, forming a long tail that is typical of hybrid semiconductors with a band gap in the visible range. In the near-infrared region (above 800 nm), the absorption becomes low, indicating weak interaction with long-wavelength light. Overall, these optical properties show that (C_5_H_7_BrN_3_)_6_(BiCl_5_)_3_ can be useful in devices that need good UV-visible light absorption.

**Fig. 10:**
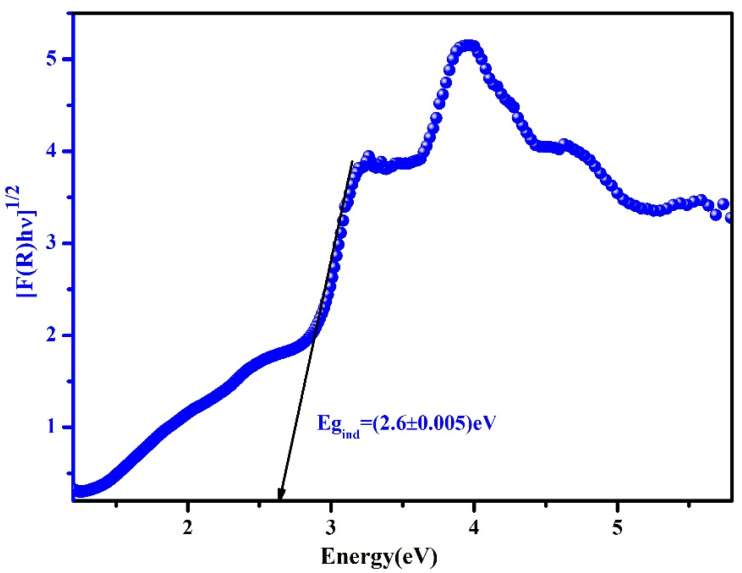
Evolution of [(*R*)*hν*/*t*]^1/2^ with the energy *hν* relative to the studied (C_5_H_7_BrN_3_)_6_(BiCl_5_)_3_ compound.

According to the Kubelka–Munk theory,^23,24^ the optical band gap of weakly absorbing materials can be estimated from diffuse reflectance measurements. The Kubelka–Munk function, defined as: 
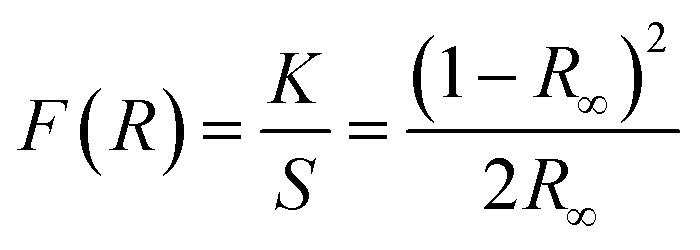
, relates the absorption coefficient *K* to the scattering coefficient *S* using the absolute reflectance *R* recorded with an integrating sphere. Based on interband transition theory,^25^ the evolution of *F*(*R*) as a function of the photon energy *hν* allows the band gap *E*_g_ to be extracted through the relationship: 
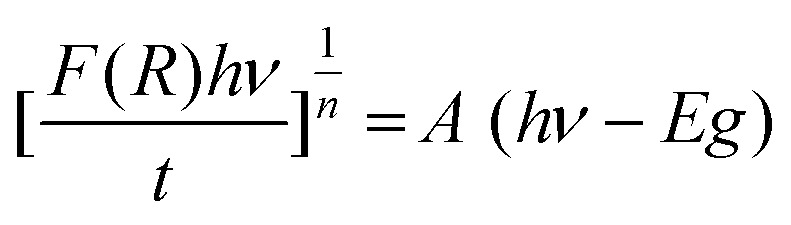
. The constant *A* is a proportionality factor related to the optical transition probability and describes the strength of light absorption in the material.^26,27^ This method is particularly suitable for bismuth-based hybrid materials, which often exhibit characteristic optical features arising from their unique electronic structure. The exponent *n* depends on the type of optical transition: it takes the value 2 for indirect band-gap transitions and 1/2 for direct ones.^28^ The plot of [(*R*)*hν*/*t*]^1/2^ as a function of *hν* is shown in Fig. 10. By extrapolating the linear portion of this curve to the photon-energy axis, the indirect band gap of the material is determined to be 2.6 eV.

**Fig. 11:**
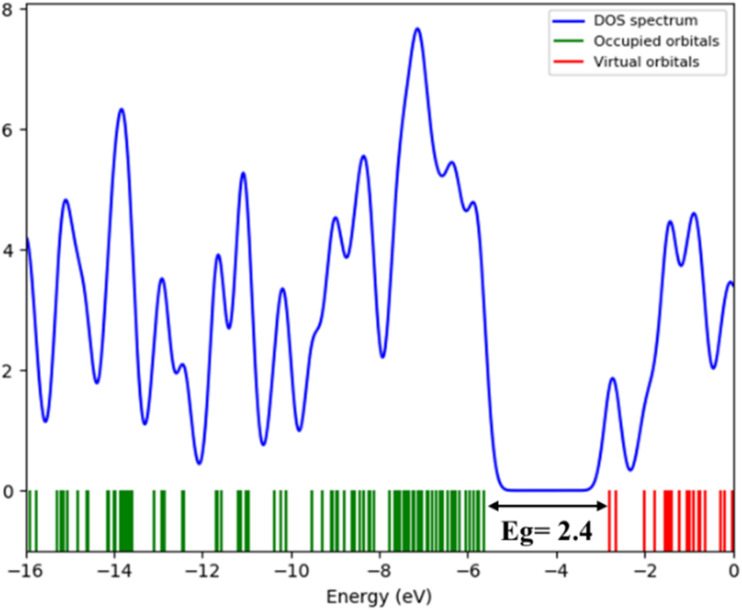
Density of state (DOS) spectrum.

**Fig. 12:**
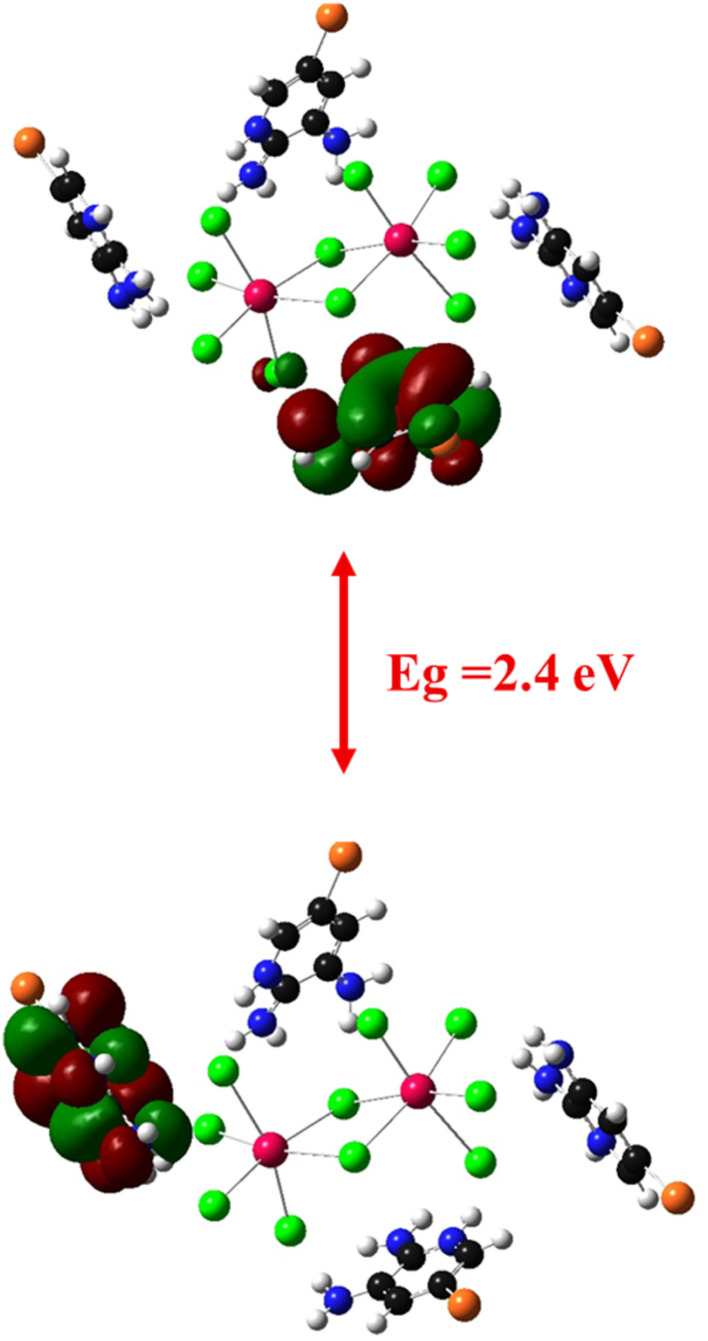
Optimized HOMO and LUMO orbitals.

This value is further supported by the density of states (Zero-DOS) analysis, which reveals a band gap of about 2.4 eV. This result aligns well with the optical measurement (Fig. 11) and confirms the semiconducting nature of the material. The calculated HOMO and LUMO orbitals (Fig. 12) further illustrate this electronic structure, showing the spatial distribution of frontier orbitals consistent with the observed band gap. The gap energy of 2.6 eV enables strong absorption within the visible region, thereby improving photovoltaic conversion efficiency. Moreover, this material shows potential for the development of visible-light-emitting diodes (LEDs), which provide versatile emission spectra and are widely used in numerous practical applications.^29^

**Fig. 13:**
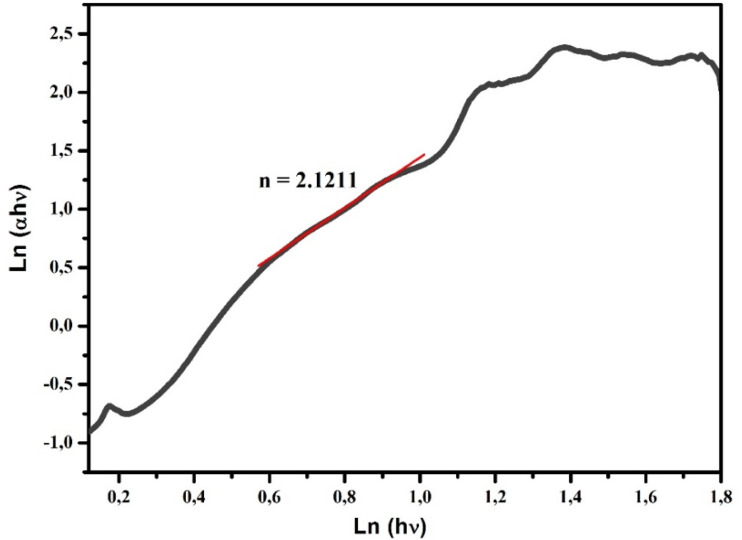
Variation of ln(*αhν*) with ln(*hν* − *E*_g_) to the studied (C_5_H_7_BrN_3_)_6_(BiCl_5_)_3_ compound.

The plot of ln(*αhν*) *versus* ln(*hν* – *E*_g_) can be used to confirm the value of the exponent *n*. As expected, the resulting curve indicates that *n* is close to 2 (2.1211), as shown in Fig. 13.

#### 3.5.2. Determination of urbach energy and threshold wavelength

**Fig. 14:**
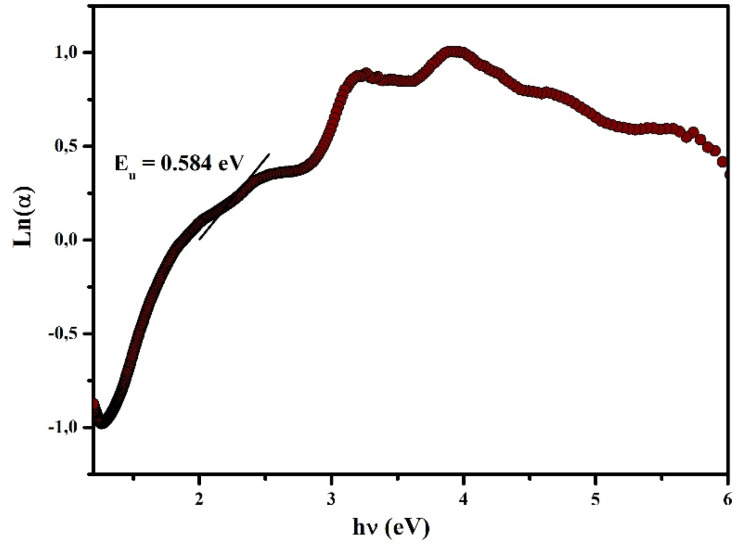
Variation of ln(*α*) as a function of *hν* for the determination of the Urbach energy *E*_u_.

The Urbach energy and the threshold wavelength offer valuable information on structural disorder and light-absorption behavior, both of which are crucial for optimizing the performance of optoelectronic materials. The *E*_u_ value can be extracted using the following relation, where *B* denotes a constant:
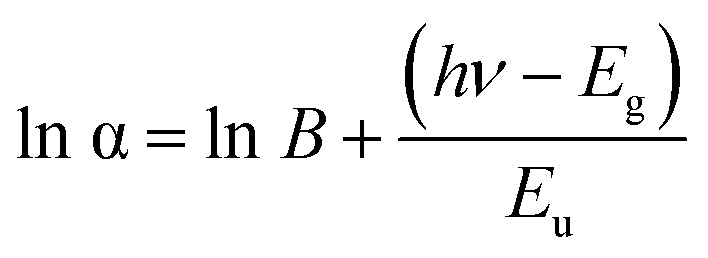


The Urbach energy (*E*_u_) was determined from the slope of the linear region in the exponential tail of the absorption edge, where ln(*α*) exhibits a linear dependence on *hν*. For (C_5_H_7_BrN_3_)_6_(BiCl_5_)_3_, the linear fitting was performed in the energy range from 2.1 to 2.4 eV, corresponding to the near-band-edge absorption region. The calculated Urbach energy is 0.584 eV, indicating a significant degree of structural disorder within the 0D bismuth-based hybrid material, which can be attributed to lattice distortions and defect-related localized states (Fig. 14). Such disorder can enhance sub-bandgap absorption, which is advantageous for broad and efficient light-harvesting applications.^30^

The maximum wavelength *λ*_i_ was determined using the following relation, in which *C* denotes a constant:^31^
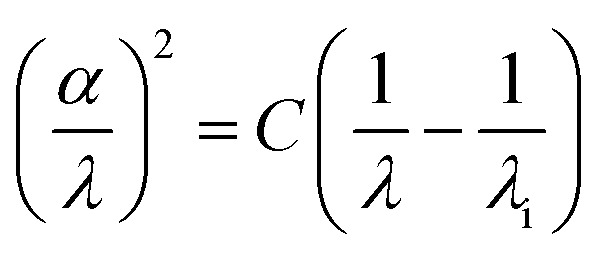


**Fig. 15:**
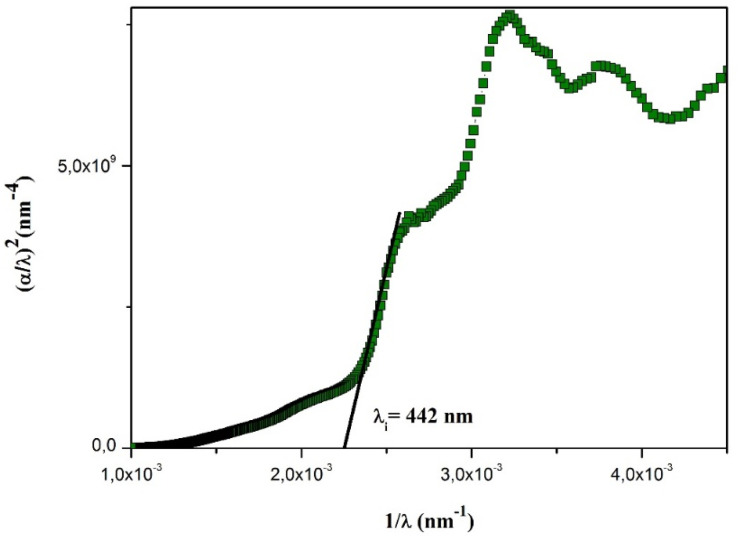
The evolution of (*α*/*λ*)^2^*versus* 1/*λ*.

The intersection of the linear fit with the baseline yields a maximum wavelength *λ*_i_ of 442 nm, which is consistent with the characteristic optical response of bismuth-based hybrid materials. This wavelength supports efficient absorption in the visible region, making the material suitable for applications such as photovoltaics and photodetectors, where effective light-harvesting and signal detection are essential (Fig. 15).

#### 3.5.3. Study of the optical conductivity

**Fig. 16:**
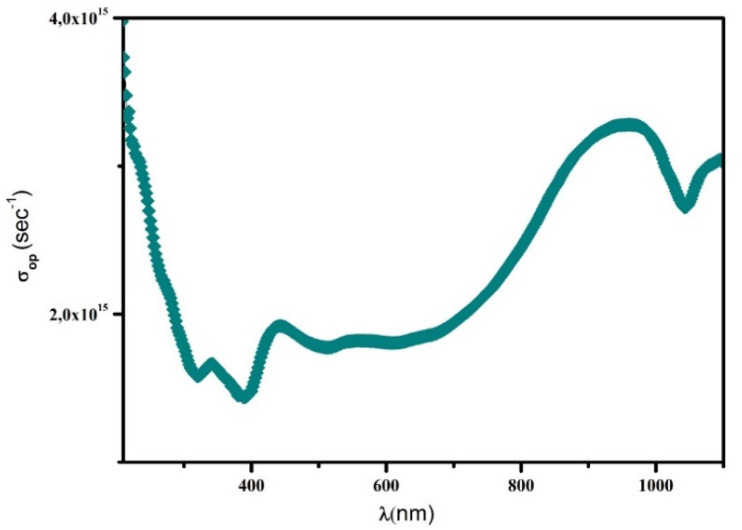
Evolution of the optical conductivity *σ*_op_ with the incident photon energy for the (C_5_H_7_BrN_3_)_6_(BiCl_5_)_3_ compound.

Optical conductivity characterizes a material's capability to transport charge carriers when exposed to light. It is directly linked to the dielectric function and to the frequency of the incident radiation. The optical conductivity *σ*_op_ can be determined using the following expression, where *c* denotes the speed of light in vacuum:
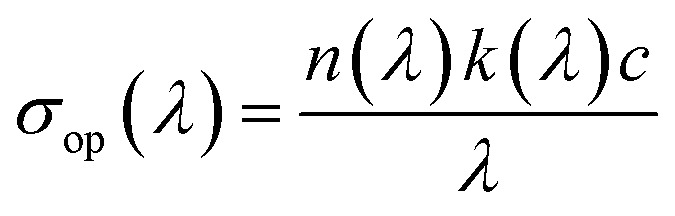
where *n*(*λ*) is the refractive index, *k*(*λ*) is the extinction coefficient, *λ* is the wavelength of the incident light, and *c* is the speed of light in vacuum. The optical conductivity curve shows how the compound reacts when it is exposed to light of different wavelengths (Fig. 16). At short wavelengths (below about 400 nm), the optical conductivity is high. This means the material absorbs a lot of light with high energy. When the wavelength increases toward the visible region (400–700 nm), the conductivity decreases and reaches a low point near 450 nm. This area corresponds to the material's band gap, where fewer electrons can be excited. After this minimum, the curve shows small up-and-down variations between 500 and 800 nm. These small changes may be caused by defects or by the structure of the material, which creates extra energy levels that can absorb light. At longer wavelengths (above 800 nm), the conductivity increases again. This suggests that the material can also absorb lower-energy light, possibly due to disorder or defect states inside the structure. In summary, the curve shows that the compound absorbs light over a wide range, from high-energy UV light to near-infrared light. This means it could be useful for devices like photodetectors or solar-energy materials.

## 4. Conclusion

The investigation of the hybrid compound (C_5_H_7_BrN_3_)_6_(BiCl_5_)_3_ has provided a comprehensive understanding of its structural organization and physical properties. The hydrothermal approach proved effective in producing a phase in which discrete inorganic units [Bi_2_Cl_10_]^4−^ and [BiCl_5_]^2−^ coexist within a lattice shaped by neutral 2,3-diamino-5-bromopyridinium molecules. The structural features revealed through powder X-ray diffraction demonstrate that the arrangement adopted in the triclinic *P̄*1 system results from a subtle balance of intermolecular interactions, highlighting the ability of the organic component to stabilize complex bismuth chloride architectures. The vibrational and electronic behaviors further support the uniqueness of this material. The IR spectrum, interpreted with the support of DFT calculations at the B3LYP/LANL2DZ level, confirms the consistency between the experimental and theoretical models. Meanwhile, the optical bandgap of 2.6 eV deduced from solid-state UV-vis measurements positions this compound within the category of indirect semiconductors, offering an interesting platform for potential optoelectronic deployment. Altogether, these findings emphasize that (C_5_H_7_BrN_3_)_6_(BiCl_5_)_3_ is not only structurally distinctive but also exhibits functional properties that merit further exploration. Future work may focus on tuning its optical response, studying its thermal or dielectric behavior, or examining its integration into advanced hybrid semiconductor systems.

## Conflicts of interest

The authors declare that they have no known competing financial interests or personal relationships that could have appeared to influence the work reported in this paper.

## Supplementary Material

RA-016-D5RA09478J-s001

## Data Availability

CCDC 2495900 contains the supplementary crystallographic data for this paper.^32^ The raw/processed data required to reproduce these findings are available and can be sent if requested.
